# Understanding Carcinogenesis for Fighting Oral Cancer

**DOI:** 10.1155/2011/603740

**Published:** 2011-05-12

**Authors:** Takuji Tanaka, Rikako Ishigamori

**Affiliations:** ^1^TCI-CaRP, 5-1-2 Minami-uzura, Gifu City, Gifu 500-8285, Japan; ^2^Oncologic Pathology, Kanazawa Medical University, 1-1 Daigaku, Uchinada, Shikawa 920-0293, Japan; ^3^Division of Cancer Development System, Carcinogenesis Research Group, National Cancer Center Research Institute, Chuo-ku, Tokyo 104-0045, Japan

## Abstract

Oral cancer is one of the major global threats to public health. Oral cancer development is a tobacco-related multistep and multifocal process involving field cancerization and carcinogenesis. The rationale for molecular-targeted prevention of oral cancer is promising. Biomarkers of genomic instability, including aneuploidy and allelic imbalance, are able to measure the cancer risk of oral premalignancies. Understanding of the biology of oral carcinogenesis will give us important advances for detecting high-risk patients, monitoring preventive interventions, assessing cancer risk, and pharmacogenomics. In addition, novel chemopreventive agents based on molecular mechanisms and targets against oral cancers will be derived from research using appropriate animal carcinogenesis models. New approaches, such as interventions with molecular-targeted agents and agent combinations in high-risk oral individuals, are undoubtedly needed to reduce the devastating worldwide consequences of oral malignancy.

## 1. Introduction

Head and neck cancer is the sixth most common human cancer [1], representing 3% of all types of cancer. They are located in the oral cavity in 48% of cases, and 90% of these are oral squamous cell carcinoma [2]. They are sometimes preceded by precancerous lesions, such as leukoplakia and erythroplakia. More than 300,000 new cases worldwide are being diagnosed with oral squamous cell carcinoma annually [3]. Approximately 35,000 new cases are recorded annually in the US [2], 40,000 new cases in the EU, and 10915 new cases in Japan [4]. The most common site for intraoral carcinoma is the tongue, which accounts for around 40% of all cases in the oral cavity proper. Tongue cancers most frequently occur on the posteriorlateral border and ventral surfaces of the tongue. The floor of the mouth is the second most common intraoral location. Less common sites include the gingival, buccal mucosa, labial mucosa, and hard plate.

The incidence of oral cancer has significant local variation. In India and other Asian countries, oral and pharyngeal carcinomas comprise up to half of all malignancies, with this particularly high prevalence being attributed to the influence of carcinogens and region-specific epidemiological factors, especially tobacco and betel quid chewing. An increase in oral cancer prevalence among young adults is a cause of special concern. There has been a 60% increase in the number of under 40 years old with tongue cancer over past 30 years. However, few data have been published on the etiology and natural history of this increase [5]. Oral malignancy including tongue cancer is associated with severe morbidity and less than 50% long-term survival despite advances in treatment (surgery, radiation, and chemotherapy) of oral cancer. The survival of the patients remains very low, mainly due to their high risk of developing a second primary cancer. Thus, early detection and prevention of oral cancer and premalignancy are quite important [6–10]. This paper will focus on our understanding of oral carcinogenesis for preventing and early detection of oral malignancy.

## 2. Oral Carcinogenesis

**Figure 1:**
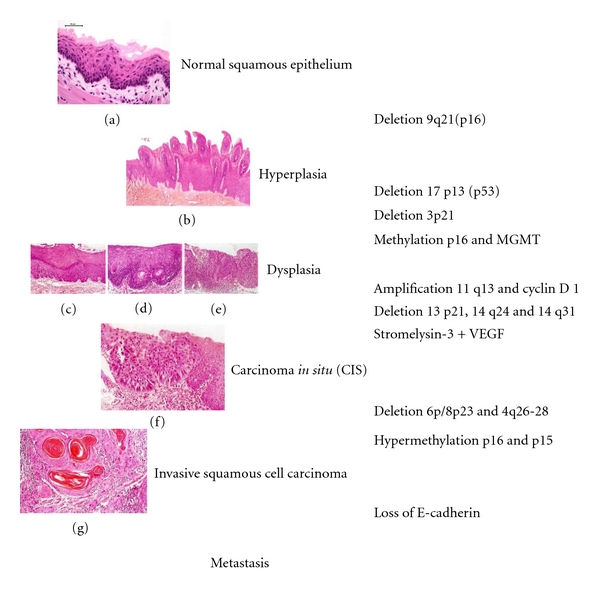
Natural history and genetic alterations of oral carcinogenesis. (a), Normal oral mucosa, (b) papillary hyperplasia, (c) midl dysplasia, (d) moderate dysplasia, (e) severe dysplasia, (f) carcinoma in situ, and (g) invasive squamous cell carcinoma (well differentiated).

Oral carcinogenesis is a highly complex multifocal process that takes place when squamous epithelium is affected by several genetic alterations. The use of several molecular biology techniques to diagnose oral precancerous lesions and cancer may markedly improve the early detection of alterations that are invisible under the microscope. This would identify patients at a high risk of developing oral cancer [11]. Natural history of oral cancer and sequence of genetic alterations are illustrated in Figure 1. There are approaches to understanding of the molecular basis of oral cancer [12–14]. They include microarray technology, methylation microarrays, gene expression microarrays, array comparative genomic hybridization, proteomics, mitochondrial arrays, and micro-RNA arrays [15]. To date, high-throughout approaches are being used to search for oral cancer biomarkers in biofluids (saliva and serum) [15].

“Field cancerization” refers to the potential development of cancer at multiple sites [16, 17]. This has been observed during the development of cancer in the tissues covered with squamous epithelium (head and neck tumor) and transitional epithelium (urothelial carcinoma). It is evident that oral cancer, like carcinomas in other tissues, develops over many years, and during this period, there are multiple sites of neoplastic transformation occurring throughout the oral cavity. Mutations of this gene have been observed in various sites of premalignant leukoplakia and carcinoma in the same oral cavity [18]. A reduction in tumor suppressor activity by the gene and the development of mutations in *p53* have been associated with smoking and an increased risk for oral carcinoma development [19]. Therefore, multi-focal presentations and mutational expressions of tumor suppressor genes may be the consequence of long-term (e.g., 20*∼*40 years) exposure to various environmental and exogenous factors. The continual presence of mutations may also signify changes in DNA repair and apoptosis, thereby increasing the susceptibility for future transformation. Mutational adaptations that modify the survivability of particular clones of transforming cells may also further enhance the level of resistance to therapeutic control. Recent genetic analysis has revealed that cancers developing at distant sites within the oral cavity often are derived from the same initial clone [20]. The multiplicity of the oral carcinogenesis process makes it difficult to interrupt the progression to cancer through surgical removal of a premalignant lesion.

## 3. Risk Factors of Oral Cancer

The most important risk factor for the development of oral cancer in the Western countries is the consumption of tobacco [21] and alcohol [22]. Although drinking and smoking are independent risk factors, they have a synergistic effect and greatly increase risk together. In Asian countries, the use of smokeless tobacco products such as gutkha and betel quid [5, 23] is responsible for a considerable percentage of oral cancer cases. Several studies have reported a significant familial component in the development of oral cancer. The estimates of risk in the first degree relatives of oral cancer patients vary widely and have been reported to be 1.1 [24] ~ 3.8 [25] although some of these refer to head and neck cancer in general. Familial aggregation of oral cancer, possibly with an autosomal dominant mode of inheritance, was reported in a very small percentage of oral cancer patients [26]. Polymorphic variation of genes in the xenobiotic metabolism pathways, such as in *CYP1A1* or the genes coding for glutathione *S*-transferase-M1 [27, 28] and *N*-acetyltransferase-2 [29] may be implicated. Individuals that carry the fast-metabolizing alcohol dehydrogenase type 3 (*ADH3*) allele [30] may be particularly vulnerable to the effects of chronic alcohol consumption and could be at increased risk to develop oral cancer [31]. 

Human papilloma virus (HPV), particularly HPV type 16, may be an etiologic factor, especially among persons who do not smoke or drink alcohol [32, 33]. Ang et al. [34] reported that tumor HPV status is a strong and independent prognostic factor for survival among patients with oropharyngeal cancer. They also noted that the risk of death significantly increased with each additional pack year of tobacco smoking. Although the idea that bacterial infections could lead to oral cancer has not been well regarded, there recently has been an increasing body of evidence to suggest a possible relationship between microorganisms and oral cancer development. The most notable example is that of the common pathogenic bacterium *Helicobacter pylori* and its association with gastric cancer. The mouth comprises a variety of different surfaces that are home to a huge diversity of microorganisms, including more than 750 distinct taxa of bacteria, suggesting that the oral squamous epithelium is constantly exposed to a variety of microbial challenges, on both cellular and molecular levels. In this context, we should draw attention to how they may relate to oral cancer development [35, 36]. 

There are clinically apparent oral premalignant lesions of oral cancer. They include leukoplakia, erythroplakia, nicotine stomatitis and tobacco pouch keratosis, lichen planus, and submucous fibrosis [37]. The term “leukoplakia” first used by Schwimmer in 1877 [38] to describe a white lesion of the tongue probably represented a syphilitic glossitis. The definition of leukoplakia has often been confusing and controversial. Some clinicians now avoid using this term. As defined by the World Health Organization, leukoplakia is “a white patch or plaque that cannot be characterized clinically or pathologically as any other disease [39]”. As such, leukoplakia should be used only as a clinical term. The term has no specific histopathological connotation and should never be used as a microscopic diagnosis. In the evaluation of the patient, leukoplakia is a clinical diagnosis of exclusion. Sometimes, a white patch is initially believed to represent leukoplakia, but the biopsy reveals another specific diagnosis. In such cases, the lesion should no longer be categorized as a leukoplakia. Leukoplakia is seen most frequently in middle-aged and older men, with an increasing prevalence with age [40]. Fewer than 1% of men below the age of 30 have leukoplakia, but the prevalence increases to an alarming 8% in men over the age of 70 [40]. The prevalence in women past the age of 70 is approximately 2%. The most common sites are the buccal mucosa, alveolar mucosa, and lower lip. However, lesions in the floor of mouth, lateral tongue, and lower lip are most likely to show dysplastic or malignant changes [41].

 The term “erythroplasia” originally used by Queyrat [42] to describe a red, precancerous lesion of the penis is used for a clinically and histopathologically similar process that occurs on the oral mucosa. Similar to the definition for leukoplakia, erythroplakia is a clinical term that refers to a red patch that cannot be defined clinically or pathologically as any other condition [39]. This definition excludes inflammatory conditions that may result in a red clinical appearance. Oral erythroplakia occurs most frequently in older men and appears as a red macule or plaque with a soft, velvety texture. The floor of mouth, lateral tongue, retromolar pad, and soft palate are the most common sites of involvement. Often the lesion is well demarcated, but some examples may gradually blend into the surrounding mucosa. Some lesions may be intermixed with white areas (erythroleukoplakia). Erythroplakia is often asymptomatic although some patients may complain of a sore, burning sensation.

 Nicotine stomatitis is a thickened, hyperkeratotic alteration of the palatal mucosa that is most frequently related to pipe smoking, but milder examples can also develop secondary to cigar smoking or, rarely, from cigarette smoking [39]. The palatal mucosa becomes thickened and hyperkeratotic, sometimes developing a fissured surface. The surface often develops popular elevations with red centers, which represent the inflamed openings of the minor salivary gland ducts.

Detection and diagnosis of oral neoplasia has traditionally relied heavily on the clinical experience of the examiners and their ability to recognize often subtle morphologic changes. However, some early malignant lesions are clinically indistinguishable from benign lesions, and some patients develop carcinomas in the absence of clinically identifiable oral premalignant lesions. Furthermore, it can be difficult even for experts to determine which oral premalignant lesions are at significant risk to progress to invasive carcinoma. Therefore, an accurate, objective, and noninvasive method to help identify premalignant lesions and to distinguish those at risk of malignant conversion is needed.

## 4. Biomarkers of Oral Cancer

Biomarkers help in the evaluation of prevention or use of therapies and the detection of the earliest stages of oral mucosal malignant transformation. Biomarkers reveal the genetic and molecular changes related to early, intermediate, and late end points in the process of oral carcinogenesis [43]. These biomarkers will refine our ability to enhance the prognosis, diagnosis, and treatment of oral carcinomas [44]. Genetic and molecular biomarkers will also determine the effectiveness and safety of chemopreventive agents. Chemopreventive agents are chemicals of natural or synthetic origin. Unlike other drugs, which do not prevent disease, chemopreventive agents reduce the incidence of diseases such as cancer before clinical symptoms occur. This development is critical for the understanding of early oral mucosal transformation. Biomarkers will also reduce the number of patients and the time for long-term follow up required to define a significant clinical response to a chemopreventive agent [45, 46]. The markers may, therefore, clarify the types, doses, frequencies, and regimens to achieve the maximum level of benefit from chemopreventive agents. Decreasing the cost of the clinical trials is another factor that drives the development of biomarkers.

 Biomarkers have been categorized following the recommendation by the Committee on Biological Markers of the National Research Council/National Academy of Sciences [47]. They fall into broad groups that detect exposure, progression, susceptibility to carcinogens, and/or the responses by the target cellular populations [46]. 

 A distinct advantage to oral cancer studies is their anatomical access to the developing premalignant and malignant lesions. One could readily analyze biopsies of the primary lesion as well as apparently normal mucosal sites to determine the levels of DNA adducts and oral cancer risk. DNA adduct studies and cytogenetic analyses may also provide evidence for altered structure and function of susceptibility sites in the DNA following DNA-binding studies of nuclear proteins such as p53. Some researchers have focused on microscopic cytogenetic and somatic mutation changes as early biologic markers. One of the markers used to define chromosomal aberrations is the staining for micronuclei in exfoliated buccal mucosal cells [48]. Micronuclei have also been used to evaluate the reversal of leukoplakia and the effectiveness of retinoids, carotenoids, and vitamin E [49, 50]. Other methods include the determination of aneuploidy, and the assessment of losses and gains of genetic material particularly associated with somatic and sex chromosomes. Other sites of chromosomal aberrations are found in sister chromatid exchanges, and allele typic variations designated by losses on chromosomes 3, 4, 5, 6, 8, 9, 11, 13, 17, and 19. 

Some molecular biomarkers with potential diagnostic relevance include DNA content and chromosome polysomy, loss of heterozygosity, nucleolar organizer regions, histo-blood group antigens, proliferation markers, increased epidermal growth factor receptor (EGFR), and decreased expression of retinoic acid receptor-*β*, p16, and p53 [51, 52]. Although a reliable, validated marker panel for providing clinically useful prognostic information in oral premalignant lesions patients has not yet been established, the advent of high throughput genomic and proteomic analysis techniques may soon yield major advances toward a prognostically relevant molecular classification system (Table 1).

## 5. Animal Models for Oral Carcinogenesis

A variety of animals has been used for the study of tumor growth, the process of carcinogenesis and the prevention/treatment research [8, 53–56]. The continual development of transgenic or knockout mice has improved our understanding of the role of specific genes in tumor growth. The most widely used animal models for oral carcinogenesis are the hamster cheek pouch model [54, 57] and the 4-nitroquinoline 1-oxide- (4-NQO-) induced oral (tongue) carcinogenesis model [8, 53, 58, 59].

In the former model, a complete carcinogen, 7,12-dimethylbenz(*a*)anthracene (DMBA, 0.5%), is applied to the hamster cheek pouch three times a week for 16 weeks. By week 16, all animals exhibit invasive oral squamous cell carcinoma. Many different studies have been conducted with the hamster buccal pouch model, and they have provided an array of changes that are analogous to those observed in human invasive oral carcinoma [54, 57]. These include a mutation in codon 61 of Ha-*ras*, which manifested in an A→T transversion in the second position of codon 61, resulting in an amino acid change from glycine to leucine. The expression of c-Ki-*ras* in malignant tumors of the pouch, but not in the normal oral mucosa, has also been observed at very early stages of tumor development [57]. Although the hamster oral tumor model appears to parallel several changes observed in human oral cancer, the hamster still has several areas of uniqueness which must be considered in any evaluations of results from oral carcinogenesis studies. The hamster cheek pouch provides a relatively large surface area of oral mucosa for the development of invasive carcinoma, while the human does not possess this type of mucosal structure. In contrast to humans, mice, or rats, the hamster cheek pouch lacks lymphatic drainage, which allows various drugs or molecules to accumulate in the pouch. The Syrian hamster population was also derived from a small breeding pair that resulted in a restricted polymorphism for the antigen recognition region (Ia region) and some of the major histocompatibility K and D regions [60]. In addition, the number of T-cells in the hamster spleen exhibits a lower number/gram weight of the organ as compared with the mouse or human [60]. The hamster may also respond to antigenic tumor sources with a natural killer macrophage or granulocyte cytotoxicity rather than a T cell response [60].

**Figure 2:**
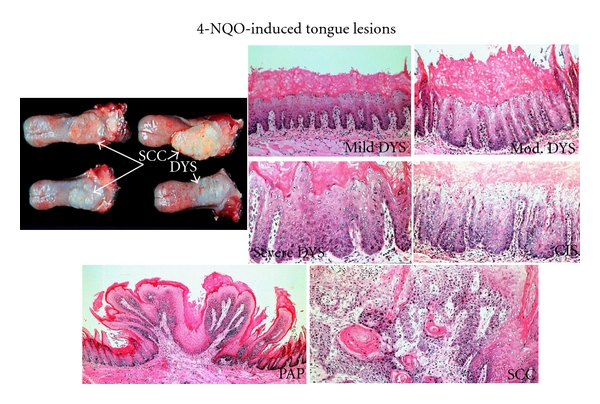
4-NQO-induced tongue lesions in rats. 4-NQO, 4-nitroquinoline 1-oxide; DYS, dysplasia; PAP, papilloma; SCC, squamous cell carcinoma.

The latter animal models for the study of oral carcinogenesis include those in rats and mice using the water-soluble carcinogen, 4-NQO. The carcinogen is either supplied in the water (20 ppm) for the rats [58, 61–74] or by painting for the mice [75]. Administration with 4-NQO in drinking water (20 ppm) for 8 weeks in rats and mice produces tongue lesions including squamous cell neoplasms (Figure 2) within 32 weeks [71], while topical application of the carcinogen to the mouse palates for up to 16 weeks, just like the hamster model develops palate tumors within 49 weeks [75]. Since the most common site for intraoral carcinoma is the tongue and the drinking water administering of 4-NQO is a simple and easy method, the 4-NQO-induced tongue carcinogenesis model is quite useful for investigating oral carcinogenesis and identifying cancer chemopreventive agents [58, 61–74, 76–84]. In the rat model, with the progression of oral carcinogenesis, increased levels of polyamine synthesis have been noted as well as nucleolar organizing regions (NORs) [58].The mouse model with 4-NQO has demonstrated some molecular mimicry of human oral cancers, as is true of the hamster model [75]. A number of chemical carcinogens including coal tar, 20-methylcholanthrene, DMBA, and 4-NQO have been used in experimental oral carcinogenesis. However, 4-NQO is the preferred carcinogen apart from DMBA in the development of experimental oral carcinogenesis. 4-NQO is a water-soluble carcinogen, which induces tumors predominantly in the oral cavity. It produces all the stages of oral carcinogenesis and several lines of evidences suggest that similar histological as well as molecular changes are observed in the human system. There are several review articles to collate the information available on mechanisms of action of 4-NQO,and studies have been carried out for the development of biomarkers and chemopreventive agents using 4-NQO animal models [8–10, 53, 58, 59, 61–68, 70–74].

The complexity and variety of biochemical changes can increase tumor development is the *p*53^−/−^ mice [85]. Unfortunately, this model and other genetic mouse models have not been exploited for studying the relationships among chemical oral carcinogenesis, specific genetic defects, and chemoprevention. Genetically altered mouse and rat models have been developed for evaluating molecular-targeted prevention and treatment of oral carcinoma [56]. We have developed *ras*H2 transgenic mouse carcinogenesis model [86] and human c-Ha-*ras* proto-oncogene transgenic rat model [87] for chemoprevention studies on oral (tongue) carcinogenesis.

## 6. Chemoprevention

Chemoprevention is the use of natural or synthetic substances to halt, delay, or reverse malignant progression in tissues at risk to develop invasive cancer [8–10]. Retinoids are the most extensively studied agents for chemoprevention of oral cancer [88]. 13-*cis*-retinoic acid given for only 3 months produced a clinical response rate of 67% versus 10% for placebo. However, toxicities were considerable, and a very high rate of relapse within 3 months of stopping treatment was reported. Subsequent studies with retinoids in patients with oral premalignant lesions have confirmed clinical and pathologic response rates though toxicities remain a concern [89]. However, translational studies showed that molecular abnormalities persisted in some patients with complete clinical and pathologic response to retinoid therapy [90], suggesting that cancer development may be delayed rather than prevented by these agents. Other agents that have been assessed in clinical trials for chemoprevention activity in oral leukoplakia patients include vitamin E [44], Bowman-Birk inhibitor concentrate (BBIC) derived from soybeans [91], curcumin [92], and green tea polyphenol epigallocatechin-3-gallate. Small clinical trials using oral BBIC revealed no significant toxicity and a 32% response rate [91].

Attention is focused now on the development of agents targeted to specific steps in the molecular progression from normal to oral premalignancy to invasive carcinoma. Examples of molecularly targeted agents that have shown promise in vitro, in animal models, or in early clinical trials include cyclooxygenase (COX)-2 inhibitors and epidermal growth factor receptor EGFR inhibitors [93–95]. Data from several sources suggest that the cyclooxygenase pathway is a good target for oral cancer prevention. COX-2 is overexpressed in head and neck squamous carcinoma [96], and COX-2 inhibitors prevented oral cancer development in animal models [97]. A randomized placebo-controlled trial of the COX-2 inhibitor ketorolac administered as an oral rinse in oral leukoplakia patients revealed that the treatment was well tolerated but did not result in greater clinical response than placebo [98]. However, analysis of the results of this trial are confounded somewhat by the high response rate (32%) in the placebo arm and difficulty in determining whether topical delivery of the agent allowed penetration to the damaged cells. The future of COX-2 inhibitors as chemoprevention agents will also depend on the determination of the extent of risk for cardiac toxicities associated with this class of agents. The EGFR is also a promising molecular target for intervention in oral malignant progression [93–95]. EGFR is a receptor tyrosine kinase that is overexpressed in oral dysplasia and invasive cancer and associated with worse prognosis in patients with head and neck squamous carcinoma [99, 100]. EGFR inhibitors, alone or in combination with chemotherapy and radiotherapy, have shown activity against head and neck squamous carcinoma in clinical trials, and toxicities were generally well tolerated [101]. Evidence has suggested that combination therapy targeting COX-2 and EGFR may be efficacious [95, 102]. Although chemoprevention appears to be a promising approach to managing oral premalignancy, prospective clinical trials using specific agents, and strong corollary translational and laboratory investigations, are needed to evaluate clinical, histologic, and molecular efficacy. In the future, it may be possible and necessary to individualize medical therapy to specific genetic abnormalities detected within the oral mucosa.

## 7. Conclusion

Human oral cancer being the sixth largest group of malignancies worldwide. Seventy percent of oral cancers appear from premalignant lesions. The process of oral cancer formation results from multiple sites of premalignant change in the oral cavity (field cancerization). Animal models are being widely used, aiming for the development of diagnostic and prognostic markers. The appearance of these premalignant lesions is one distinct feature of human oral cancer. At present, there is dearth of biomarkers to identify which of these lesions will turn into malignancy. Regional lymph node metastasis and locoregional recurrence are the major factors responsible for the limited survival of patients with oral cancer. Paucity of early diagnostic and prognostic markers is one of the contributory factors for higher mortality rates. Determining high- and low-risk populations by measuring reliable biomarkers help us to understand the dynamics and prevention of oral cancer development. The quantitation of genetic and molecular changes and the use of these changes as markers for the detection and prevention of early premalignant change require the harvesting of tissues and cells. Promising technologies are being rapidly developed to assist in localization of abnormal oral mucosa, in noninvasive and objective diagnosis and characterization of identified mucosal lesions, and in therapy of patients with oral cancer. Undoubtedly, the prevention or reduction in the smoking of tobacco products and alcohol consumption would have a profound influence on the incidence of oral cancer. Chemoprevention also has an impact on the development of malignant changes in the oral mucosa. Prevention through chemoprevention and/or the use of systemic medications has been an extensively studied strategy and continues to hold promise as a way of diminishing the morbidity and mortality associated with this malignancy.

## Figures and Tables

**Table 1:** Potential biomarkers for oral carcinogenesis.

Category	Measures
Genomic biomarker	Micronuclei, DNA adduct, DNA content, and chromosomal aberration (polymorphism, alleic loss, gain, and amplification)
Oncogenic biomarker	Oncogenic expression, modified tumor suppressor genes, and *Src* genes
Proliferation biomarker	Nuclear and cyclin-related antigens, mitotic frequency, ornithine decarboxylase (ODC), and polyamines
Differentiation biomarker	Cytokeratins, transglutaminase Type I, and transcription factor (AP)-1
Oxidative stress biomarker	Glutathione *S*-transferase, stress proteins (HSPs), and Superoxide dismutase
Apoptosis biomarker	Bcl-2 family, chromatin condensation factors, caspases, and nucleosome formation
Immunologic biomarker	Cytokines

## Abbreviations

BBIC: Bowman-Birk inhibitor concentrate
COX: Cyclooxygenase
DMBA: 7,12-dimethylbenz(*a*)anthracene
EGFR: Epidermal growth factor receptor
IL: Interleukin
4-NQO: 4-nitroquinoline 1-oxide.
